# Automatic Speech Recognition Performance Improvement for Mandarin Based on Optimizing Gain Control Strategy

**DOI:** 10.3390/s22083027

**Published:** 2022-04-15

**Authors:** Desheng Wang, Yangjie Wei, Ke Zhang, Dong Ji, Yi Wang

**Affiliations:** Key Laboratory of Intelligent Computing in Medical Image, Ministry of Education, School of Computer Science and Engineering, Northeastern University, Shenyang 110169, China; deshengwang001@gmail.com (D.W.); 1910621@stu.neu.edu.cn (K.Z.); jidong@cse.neu.edu.cn (D.J.); wangyi@cse.neu.edu.cn (Y.W.)

**Keywords:** human–computer interaction, automatic speech recognition (ASR), word error rate (WER), gain control, noise figure, maximized original signal transmission (MOST)

## Abstract

Automatic speech recognition (ASR) is an essential technique of human–computer interactions; gain control is a commonly used operation in ASR. However, inappropriate gain control strategies can lead to an increase in the word error rate (WER) of ASR. As there is a current lack of sufficient theoretical analyses and proof of the relationship between gain control and WER, various unconstrained gain control strategies have been adopted on realistic ASR systems, and the optimal gain control with respect to the lowest WER, is rarely achieved. A gain control strategy named maximized original signal transmission (MOST) is proposed in this study to minimize the adverse impact of gain control on ASR systems. First, by modeling the gain control strategy, the quantitative relationship between the gain control strategy and the ASR performance was established using the noise figure index. Second, through an analysis of the quantitative relationship, an optimal MOST gain control strategy with minimal performance degradation was theoretically deduced. Finally, comprehensive comparative experiments on a Mandarin dataset show that the proposed MOST gain control strategy can significantly reduce the WER of the experimental ASR system, with a 10% mean absolute WER reduction at −9 dB gain.

## 1. Introduction

Automatic speech recognition (ASR) has been widely integrated into human–robot interactions in the form of voice user interfaces (VUIs) [1,2,3]. Virtual assistants [4], vehicle systems [5], and home automation all make daily life more convenient [6,7,8,9], and the application scope of ASR is growing in popularity as more people have recognized VUIs as more natural than graphical user interfaces (GUIs) [10,11].

Currently, the performance of the ASR system in many human–robot interaction scenarios is unsatisfactory due to robustness limitations, and one of the critical factors is that various practical noises make it more challenging to extract the features, such as Mel-frequency cepstral coefficients (MFCC) [12,13,14], log-channel energies [15], and pitch-based features [12,16]. Some common noises have been widely researched by experts in ASR, such as background noise [9,17], reverberation [18,19,20,21], squeal noise, and noises tightly related to hardware, such as thermal noises from amplifiers [22], quantizing noises from analog to digital converters (ADCs) [23], and signal quality loss caused by coding [24], compression, and transmission [25]. However, noises related to gain controls have received less attention. Gain control represents the amplitude adjustment of signals, and it is one of the frequently used operations in ASR systems. A large gain may cause the ASR system not to work properly, such as data overflow from the software perspective, and clipping from the hardware perspective. Therefore, gain control in this paper refers to original gain controls under the premise of no clipping occurring.

**Figure 1:**
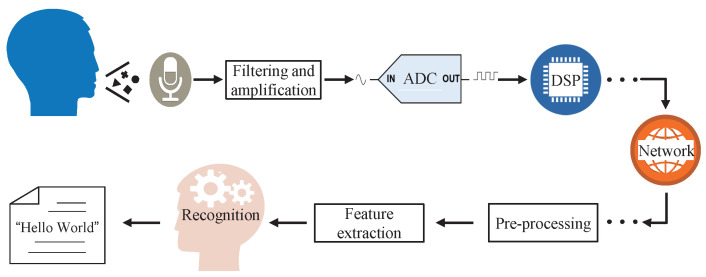
General audio signal flow of the ASR deployed in the human–computer interaction system.

Figure 1 represents a typical signal flow diagram of the ASR system deployed in a human–robot interaction system. Speech signals go through multiple gain controls of serial function units before being processed by the recognition module. Generally, the function units near the user-end include microphone(s), anti-aliasing filtering and dynamic range adjustment, analog to digital converter (ADC), and basic digital signal processing (DSP), such as enhancement, denoising, audio coding, and decoding. Function units in the cloud include pre-processing, feature extraction, and recognition. The speech signals are transmitted to the cloud though the network.

**Figure 2:**
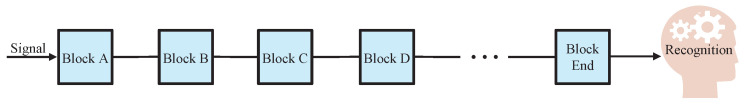
Gain control distribution within the audio signal flow of the ASR system.

The function units before the recognition in Figure 1 are further abstracted as serial blocks in Figure 2 to illustrate the gain control issue. The gain control operations are distributed in these blocks in Figure 2. The gain within each block is called the gain requirement. The gain control strategy in this paper refers to the gain distribution while performing the gain control operations. Constrained gain controls refer to gain distributions that conform to certain rules, and unconstrained gain controls mean free gain distributions. Assume the gain requirement from “Block D” is −3 dB. The gain distribution can vary a lot, such as follows. (1) The gain of −3 dB is divided as −1 dB, −1 dB, and −1 dB on “Block A”, “Block B”, and “Block C”, respectively; (2) The gain of −3 dB is divided as −2 dB, −1 dB, and 0 dB on “Block A”, “Block B”, and “Block C” respectively; (3) The gain of −3 dB is only performed on “Block C”.

From the perspective of noise, theories in [26] show that the noises caused by gain control can indirectly affect the accuracy of recognition by distorting the features of speech signals. However, even for a fixed gain value, different gain control strategies correspond to different noise levels, distorting the features of speech signals to different degrees. Therefore, to improve the performance of ASR systems, it is necessary to optimize the gain control strategy in ASR or ASR-related research [18,27,28].

This paper proposes an optimal gain control strategy named the maximized original signal transmission (MOST) to minimize the adverse effect of noises induced by gain control on ASR. The gain control strategy within the ASR system can be optimized, since it has been shown that the performances of similar systems, such as RF systems, can be significantly improved by modifying the gain structures [26,29]. Firstly, the gain control strategy’s influence on the speech signal quality is analyzed by establishing a general model of the ASR system’s gain control strategy. Secondly, based on the established model using the noise figure theory from the radio frequency (RF) area, the proposed MOST gain control strategy is proved optimal from the aspect of the gain control strategy. Thirdly, for complex and diverse ASR systems in practice, a general implementation framework of the proposed MOST gain control strategy is given, which realizes the proposed MOST gain control strategy by automatically managing gain control logic and operations. Finally, the effectiveness of the proposed MOST gain control strategy is verified on the experimental ASR system for Mandarin.

The paper is organized as follows: In Section 2, related works in the literature and the motivation are briefly introduced. In Section 3, the modeling analysis and proof of gain control influence on ASR systems are given, and the proposed MOST gain control strategy and the corresponding implementation framework are presented accordingly. In Section 4, experiments based on the critical factors of reverberation and noises that influence the WER of ASR systems are carried out on our hardware platform. The conclusions are summarized in Section 5.

## 2. Related Work

Currently, most of the research on ASR focus on recognition-related algorithms, and there is insufficient research on gain control strategies that actually have a significant impact on ASR systems. The recognition-related algorithms on ASR include two main categories: the pre-processing algorithms and machine learning (ML)-based ASR algorithms. The pre-processing algorithms, such as dereverberation [18,19,20,21] and denoising [30], usually greatly promote the ASR system’s performance. Typical pre-processing methods are beamforming methods based on microphone arrays [31]. For the ML-based ASR algorithms, there are various kinds of neural network-related research studies [12,32,33], whose architectures generally involve the artificial neural network (ANN) [34], deep convolutional neural network (DNN) [33], recurrent neural network (RNN) [35], fuzzy neural network (FNN) [32], etc. The acoustic model (AM), pronunciation model (PM), and language model (LM) are primary aspects that the ML-based ASR algorithms need to consider. However, AM, PM, and LM can be folded into a single network for joint training by using a sequence-to-sequence model [36]. The recently proposed ASR approach in [14] achieves a speedup of about 50 times over the comparison method by combining the end-to-end model with the non-autoregressive speech recognition model. Moreover, ASR for second language pronunciation training and learning is currently a hot topic [37]; for example, research on pronunciation assessment of L2 Spanish for Japanese speakers [38].

In ML-based ASR algorithms, the recognition process works on the extracted features. As shown in the lower part of Figure 1, the function unit of feature extraction is in front of the recognition. Moreover, gain controls are widely integrated to realize functions, such as adjusting the signal strength [7,17,27], improving the perceptual intelligibility [8,9], optimizing the ASR performance directly [28], or by speech enhancement [18,19,39,40].

**Figure 3:**
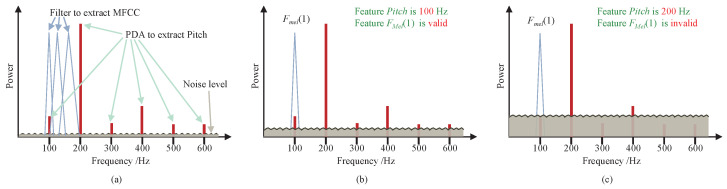
Noise influence on MFCC and pitch features. (**a**) Clean speech signal. (**b**) Speech signal with low level noise. (**c**) Speech signal with high level noise. PDA is the pitch estimation algorithm.

However, the noises caused by gain controls distort the features, such as Mel-cepstral features [14] and pitch-based features [12,16]. From the perspective of frequency-domain, these features are directly or indirectly constituted by the harmonics of the speech signals. Figure 3 illustrates a short time Fourier transform (STFT) of a frame of speech signal, the harmonics located within the first Mel-filter characterize the first dimension of the MFCC feature $f_{Mel}\left(1\right)$, and the first few significant harmonics together determine the pitch. The noise levels corresponding to different gain control strategies may lead to quite different feature extraction results. As the noise level increases, the harmonic components are gradually flooded, such as the 1th , 3th , 5th, and 6th harmonics in Figure 3c. The pitch feature in Figure 3b keeps the same with that in Figure 3a [41,42]. However, an error pitch occurs in Figure 3c. Similarly, the feature $f_{Mel}\left(1\right)$ in Figure 3b is valid while it is invalid in Figure 3c. Thus, an optimized gain control strategy with a lower noise level reserves more features of speech signals, and this could be helpful to reduce the WER of ASR.

## 3. Proposed Gain Control Strategy and Modeling Analysis

In this section, the MOST gain control strategy is first proposed, and then the detailed modeling and analysis for deriving the proposed MOST gain control strategy are demonstrated. For the convenience of the subsequent description, the components or subsystems within the ASR system are classified into three categories: (1) receiving unit, (2) middle unit, and (3) recognition unit. For the ASR system shown in Figure 1, the microphone is the receiving unit, the ASR algorithm is the recognition unit, and all other components or subsystems are regarded as the middle units.

### 3.1. Proposed Gain Control Strategy

The proposed MOST gain control strategy is a constraint on gain controls: gain control operations are performed as close to the recognition unit as possible. In practice, the constraint as close to the recognition unit as possible must be considered in the following two perspectives. For clarity, assume the ASR system in the listed cases all consist of a receiving unit, three middle units, and a recognition unit.

- From the perspective of different units, assume that a gain of −3 dB is required in the recognition unit. For the current gain control strategy, the execution position of the gain of −3 dB is unconstrained, and it can be performed at the output of the receiving unit, or −1 dB at each middle unit, etc. On the contrary, the proposed MOST gain control strategy means that the −3 dB gain must be performed at the output of the last middle unit;
- From the perspective of a specific unit, assume that the middle unit of pre-processing needs a −2 dB gain control. Ensuring the −2 dB gain control is only performed on this middle unit does not satisfy the constraint. Because this middle unit probably consists of many smaller software function units, the gain control also has an execution order among these smaller function units. Thus, the −2 dB gain control should be placed as close to the end of these smaller function units.

**Figure 4:**
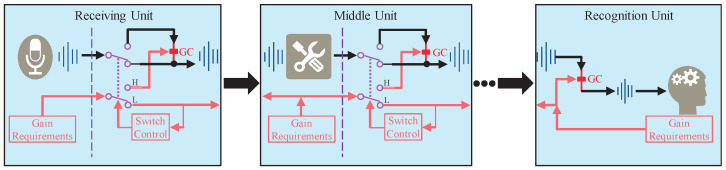
Proposed MOST gain control strategy for the ASR system.

The name MOST alludes to the optimization effect and the optimization method. The effect of the proposed gain control strategy lies in the maximum preservation of the speech signal features, and the description “maximum original signal transmission” represents the idea of the proposed gain control strategy. The proposed MOST gain control strategy can be implemented in many specific forms in practice, such as presented in Figure 4. The audio signals are transmitted in the direction of the upper black arrows. The red arrows represent the direction of the gain control command and the logic of the gain control operation. The middle part of the receiving and middle units are two double pole double throw switches. “GC” represents gain control operation, “H” and “L” represents the “switch control” corresponding to the control logic when the acknowledgement (ACK) signal is received or not, respectively. The points between the middle unit and the recognition unit represent the middle units. The three gray icons on the left part of the receiving and middle units, and in the right part of the recognition unit, represent the functions of these units. They are receiving, processing, and recognition, respectively.

The advantage of the form in Figure 4 is that it provides a compatible protocol framework to establish communication between different units, thereby providing support for the proposed MOST gain control strategy. In detail, the signal from the receiving unit is transmitted and processed by several middle units, and finally reaches the recognition unit. The proposed MOST gain control strategy in Figure 4 consists of two parts:

- Transmission channel for gain control commands represented by the arrow between units, which can be wired or wireless, together or independently;
- Gain control logic on the right side of the dotted line in each unit. The double pole double throw is critical, because it maps the switch control command into the corresponding gain control logic.

When different units are connected, the units with the proposed MOST gain control strategy perform two actions before establishing the transmission channel for gain control commands.

- Wait for a handshake signal at the input. If the handshake signal is received, return the ACK signal to the former unit;
- Send handshake signals periodically on the output and check for the ACK signal. If received, return the ACK signal to the switch control module.

The two actions are used to establish communication between adjacent units. Wait for a handshake signal is attempting to establish communication between the current unit and the former unit. Send handshake signals periodically is attempting to establish communication between the current unit and the latter unit.

### 3.2. Modeling of Gain Control in ASR Systems

The modeling and analysis of gain control strategies in ASR systems are achieved by the following two steps. (1) Establish the gain control strategy model by regarding the macroscopic function units in ASR systems as the microscopic components in the RF signal chain; (2) Analyze the gain control strategy model using the noise figure theory.

**Figure 5:**
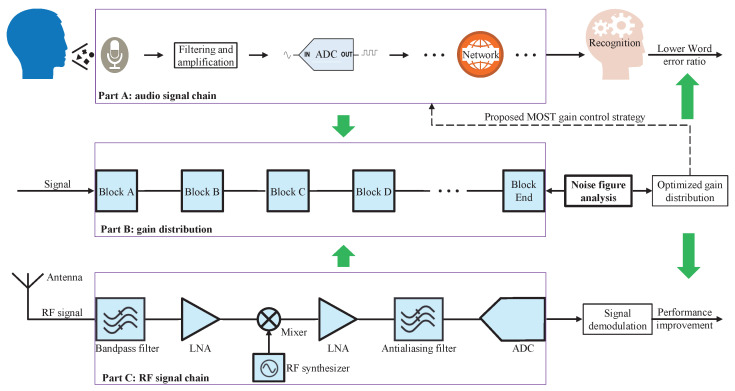
Principles of modeling and analysis of gain control strategy.

Figure 5 shows the details of the modeling and analysis, wherein “Part A” represents the general block diagram of the ASR systems’ signal flow. “Part B” illustrates the abstracted serial blocks used to model and an analysis of the gain control strategy. “Part C” is a generic block diagram of the transceiver’s RF signal chain. The RF signal chain in “Part C” is composed of several components in series, such as a low noise amplifier (LNA), a diode, or the like. The gain distributions of these components severely influence the subsequent signal demodulation. The noise figure theory is an effective way at optimizing the influence of gain control because it establishes the relationship between gain distribution and signal quality [26]. Thus, the gain control strategy in “Part A” can be optimized as long as “Part A” is abstracted as the form of “Part B”. The following will first introduce the noise figure theory and then demonstrate how to abstract a general model of the gain control strategy from diverse ASR systems in practice.

Noise figure precisely reflects the influence of the components’ cascaded gain and distortion on the signal chain quality [26]. Specifically, noise figure represents the degradation of the signal to noise ratio (SNR) when a signal goes through a device [26,29]. Noise figure *F* is defined as (1), where ${S\;N\;R}_{\mathrm{input}}$ is the input SNR, ${S\;N\;R}_{\mathrm{output}}$ is the output SNR. Parameters ${S\;N\;R}_{\mathrm{input}}$ and ${S\;N\;R}_{\mathrm{output}}$ generally refer to the ratio of the signal component and the noise component at the hardware level. Thus, the value of noise figure is always no less than 1. In particular, the noise figure value of an ideal system causing no distortion is 1, and a value closer to 1 indicates a better system performance.

(1) $$F=\frac{{S\;N\;R}_{\mathrm{input}}}{S\;N\;R_{\mathrm{output}}}$$

The RF chain is a cascade formed by components and sub-units. By equating the units of the ASR system as components in the RF chain, the noise figure index is used to analyze the gain control strategy in this paper. Assume the gain of an ASR system in logarithmic form is $G_{\mathrm{req}}$ dB, the corresponding gain in linear form $GL_{\mathrm{req}}$ is

(2) $$GL_{\mathrm{req}}=10^{\left(\frac{G_{\mathrm{req}}}{20}\right)}$$

An ideal gain control attenuates both the power of the speech signal and the noise by the same degree, which is assumed as $G_{\mathrm{req}}$. Thus, the noise figure of the ideal gain control can be derived

(3) $$F_{\mathrm{ideal}}=\frac{\frac{P_{\mathrm{signal}}}{P_{\mathrm{noise}}}}{\frac{P_{\mathrm{signal}}\times {G_{\mathrm{req}}}^{2}}{P_{\mathrm{noise}}\times {G_{\mathrm{req}}}^{2}}}=1$$

where $P_{\mathrm{signal}}$ and $P_{\mathrm{noise}}$ denote the power of the signal and the noise, respectively. Differently, the noise floor of the actual gain control is generally fixed and determined by inherent characteristics, such as resolution. Assume the power of this noise floor is $P_{\mathrm{noisefloor}}$, the corresponding noise figure is

(4) $$F_{\mathrm{actual}}=\frac{\frac{P_{\mathrm{signal}}}{P_{\mathrm{noise}}}}{\frac{P_{\mathrm{signal}}\times {G_{\mathrm{req}}}^{2}}{P_{\mathrm{noisefloor}}}}>1$$

Equation (4) shows that speech signal quality degradation is unavoidable if the gain control operation is used. Thus, it is of significance to minimize the impact of the gain control operation, which corresponds to minimizing the value of the noise figure. Next, to achieve this goal, we discuss how to perform the optimal gain control strategy through a modeling analysis.

**Figure 6:**
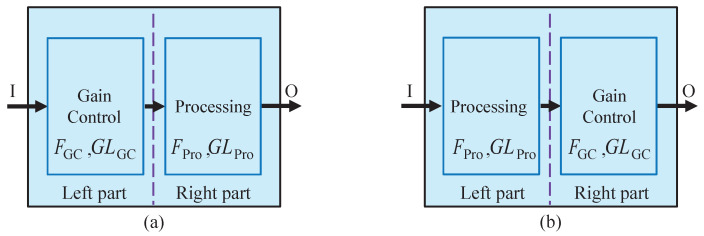
Basic unit models that constitute the gain control strategy. (**a**) Gain control is before the processing portion. (**b**) Gain control is after the processing portion.

The general gain control strategy model of ASR systems is established by modeling the basic unit mentioned in Figure 4. While modeling the unit, it is specified that, if a unit contains the gain control, the gain control is at the input or output position of the unit. Otherwise, the unit continues to be split, and replaced by smaller units. Therefore, any unit can be equivalent to the mixture of the gain control and the processing portion. For a certain unit, if these two parts do not exist simultaneously, the missing part can be equivalently added by an all-pass function with the noise figure and the linear gain both equal to 1. Thus, the basic unit models that constitute the gain control strategy are shown in Figure 6.

The overall gain of the ASR system with the unit model number of *n* is the combination of all the units

(5) $$GL_{\mathrm{overall}}=\prod_{i=1}^{n}GL_{{\mathrm{Pro}}_{i}}GL_{{\mathrm{GC}}_{i}}$$

Assume that the ASR system consists of *n* basic unit models; that is, the ASR system includes *n* processing portions and *n* gain controls. The noise figure of the ASR system can be calculated by treating each macroscopic processing portion and gain control as microcosmic components in an RF chain, and by applying the noise figure formula of the cascade system

(6) $$\begin{array}{cc} F_{{A\;S\;R}_{n}} & =F_{1a}+\frac{F_{1b}-1}{GL_{1a}}+\frac{F_{2a}-1}{GL_{1a}GL_{1b}}+\frac{F_{2b}-1}{GL_{1a}GL_{1b}GL_{2a}}+\cdot \cdot \cdot \\ \; & +\frac{F_{c}-1}{GL_{1a}GL_{1b}GL_{2a}\cdot \cdot \cdot GL_{c-1}}+\cdot \cdot \cdot \frac{F_{nb}-1}{GL_{1a}GL_{1b}GL_{2a}GL_{2b}\cdot \cdot \cdot GL_{na}} \end{array}$$

where parameters *F* and GL with the index 1a, 2a, …represent the noise figure and the gain of the left part of the basic unit model in Figure 6a or Figure 6b, respectively. Similarly, parameters *F* and GL with the index 1b, 2b, …represent the noise figure and the gain of the right part of the basic unit model in Figure 6a or Figure 6b, respectively.

### 3.3. Analysis and Proof

The proposed MOST gain control strategy constrains each gain control requirement to be performed as close as possible to the end of the signal flow. Although there may be multiple gain control requirements from the units within the ASR system, the analysis and proof that the proposed MOST gain control strategy is optimal is applicable to all gain control requirements. Select any one of the gain control requirements. Assuming that the value of the selected gain control requirement is $GL_{\mathrm{sel}}$ produced by the *k*th basic unit model, and the unit’s function limits that the selected gain control requirement can be moved backward in the signal flow by, at most, *m*th units, and is performed on the (k+m)th unit, m≥ 0.

The argument is the influence of the gain control strategies, so the processing induced by the last recognition unit itself should not be considered. Since the method involves comparing the overall noise figure value of ASR systems with different gain control strategies, it is reasonable and necessary to set the last recognition unit that does not affect the conclusion as an all-pass function. By doing so, the influence of the gain control strategy on ASR systems is reflected by the value of the noise figure. The minimum noise figure corresponds to the optimal system performance. Thus, proving that the proposed MOST gain control strategy is optimal is equivalent to proving that the overall noise figure of the ASR system corresponding to the gain control requirement is only performed in the *m*th unit is the smallest.

$GL_{\mathrm{GC}}$ influences the overall noise figure of the ASR system $F_{{A\;S\;R}_{n}}$ through (6). Because different gain control strategies contribute differently to each of the cumulative terms in (6), the ASR system’s overall noise figures of the current strategies and the proposed MOST gain control strategy are different. The fixed values of $F_{\mathrm{Pro}}$ and $GL_{\mathrm{Pro}}$ do not affect the analysis of gain control strategy. Thus, the analysis and proof only need to consider the gain distribution, rather than the $F_{\mathrm{Pro}}$ and $GL_{\mathrm{Pro}}$. The reason is that the noise figure $F_{\mathrm{Pro}}$ and the gain $GL_{\mathrm{Pro}}$ of each unit are both fixed and determined by the performance and function of the processing portion, respectively, such as the gain $GL_{\mathrm{Pro}}$ of the analog processing circuit between the microphone and ADC. The gain $GL_{\mathrm{Pro}}$ is generally constant and depends on the signal amplitude difference between the microphone output and the ADC input, respectively.

According to the gain distribution of the gain control requirement $GL_{\mathrm{sel}}$ expressed by (5), for the proposed MOST gain control strategy, $GL_{\mathrm{GC}}$ = 1 for *i* = 1,2,…m−1, and $GL_{\mathrm{GC}}$ = $GL_{\mathrm{sel}}$ for *i* = *m*. Moreover, for the current gain control strategy, usually at least one $GL_{\mathrm{GC}}$ < 1 for *i* = 1,2,…m−1, and $GL_{\mathrm{GC}}$ < $GL_{\mathrm{sel}}$ for *i* = *m*. Thus, for the selected gain control requirement, the comparison of the overall noise figure of the ASR system under the current gain control strategy and the proposed MOST gain control strategy can be achieved by letting *m* equal to *n* in (6), respectively. Assume that, in the above-mentioned, at least one gain $GL_{\mathrm{GC}}$ < 1 in the current gain control strategy is indexed by *c* in (6). Moreover, this gain is equal to 1 in the proposed gain control strategy. Since the gains in (6) are at the numerator position, the larger the gain, the closer to the former position, the smaller the corresponding noise figure. As a result, the overall noise figure of the current gain control strategy must be greater than that of the proposed MOST gain control strategy. The proposed gain control strategy minimizes the overall noise figure by maximizing the numerators of the first m−1 terms in (6). Thus, the proposed gain control strategy is an optimal gain control strategy.

## 4. Experiment

The experiment was set up to compare the ASR system’s performance of current gain control strategies and our proposed MOST gain control strategy.

Since the gain control strategy of practical ASR systems is diverse, even under the same overall gain setting, gain control strategies are uncertain, because the position, number, and allocation proportion of the gain control all could be varied. Therefore, it is of great significance to cover various actual situations by elaborately designing a limited set of experiments. Section 3 theoretically demonstrates that the influence of gain control is dependent on the gain distribution within the signal flow, and the proposed MOST gain control strategy adds a constraint that the gain control operation is performed only at the last recognition unit. Therefore, the theoretical analysis can be verified by comparing the ASR system performance under extreme gain control configurations (performed at two ends, respectively).

### 4.1. Experimental Setup Overview

In the experiment, we selected a practical and widely used ASR scenario. The ASR scenario was a smart voice recorder that worked in actual environments. Noise and reverberation are two significant factors affecting ASR systems [6,20,21]. The actual environments were designed to be comprehensive and representative by including various types of noises, various SNRs, various reverberation strengths, and various speaker to microphone distances. The advantage of the proposed gain control strategy was manifested by the WER reduction of ASR.

The voice recorder’s ASR system included two parts: the user-end device and the processing in the cloud. The two parts were connected through a network. The user-end device received and converted the sound into an electrical signal. After preprocessing, such as filtering and analog to digital converting, the digital audio signal was transmitted to the cloud through the network to achieve the text transfer function by the recognition algorithm. This practical ASR system was equivalently simulated using a wireless audio transmission system developed by us and two computers for comparison experiments.

**Figure 7:**
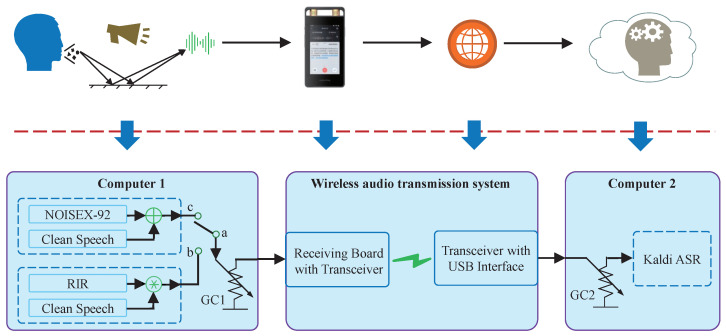
ASR experiment block diagram and the corresponding relationship between the experiment setup (**below the dotted line**) and the practical ASR system (**above the dotted line**).

The block diagram of the ASR experiment is illustrated in Figure 7, which includes two utterance generation modules on computer 1, the wireless audio transmission system, and an ASR module on computer 2. We assumed that the gain control operation was only performed on “GC1” or “GC2” in the current strategies or the MOST gain control strategy, respectively. Two utterance generation modules based on the noise and reverberation were designed to comprehensively compare the current and the proposed gain control strategies. The utterance generation modules and the ASR module were on two independent computers.

The corresponding relationships between the voice recorder with text transfer function and the ASR experiment are explained as follows.

- The test utterances were generated on computer 1 to make the reverberation degree and noise level of the experimental speech signal more controllable and quantitatively modified;
- The wireless audio transmission system contained a receiving board and a transceiver with a USB interface; the details are in Appendix A. The user-end device corresponded to the receiving board, within which, the filtering and other processing were integrated to simulate the actual noises induced by the hardware. The wireless transceiver was designed to simulate the actual network transmission of the voice recorder;
- The ASR function in the cloud was simulated through a locally installed Kaldi ASR module on computer 2.

#### 4.1.1. ASR Module and Dataset Selection

The proposed gain control strategy optimizes the speech signal quality; therefore, the WER improvement effect of the proposed gain control strategy is universal for ASR systems. This paper selects the open-source ASR toolkit Kaldi [43] and the Mandarin TDNN chain model CVTE trained on commercial data as the experimental ASR module. The dataset in this experiment was part of the test set of THCHS-30 [44], which contained 500 test utterances recorded from 10 native speakers, including males and females. These speakers’ ages were from 19 to 50, all of them were fluent in standard Mandarin. The sampling frequency of the test utterances was 16 kHz, and the resolution was 16-bit. The WER results are the statistical averaged values of these test utterances.

#### 4.1.2. Noise Setup

The utterance generation module with noises was enabled by switching on the “a” and “c” in Figure 7. The white, babble, and factory1 noise in the NOISEX-92 noise library [45] were attenuated and then superimposed on the clean speech according to the SNR, respectively. Since the sampling frequency of the noise dataset was not consistent with the sampling frequency of the clean speech dataset, the noises were resampled. The resample process was realized by the commonly used resample function (default parameters setup) within the MATLAB tool. The resample function adopted a linear interpolation and an anti-aliasing filter to resample the signal at a uniform sample rate. The cutoff frequency of the anti-aliasing filter was set to the Nyquist frequency of the lower sample rate (here, it was 16 kHz). The anti-aliasing filter is a linear-phase FIR filter with the Kaiser window (β = 5). In actual environments, the power of the audio signal received by the microphone decreases rapidly as the speaker-microphone distance increases. We simulated this by adding an attenuator after the clean speech; the attenuator before the noise signal was set accordingly at the same time to achieve the test utterances with a specified SNR.

#### 4.1.3. Reverberation Setup

The utterance generation module with reverberations was enabled by switching on the “a” and “b” in Figure 7. The severity of room reverberation was quantified by the reverberation time and reverberation time 60 (RT60) was widely used in practice. RT60 is the time it takes for a sound to decay by 60 dB. A higher RT60 represents a more severe reverberation. Commonly used RT60s of 0.5 s, 0.7 s, and 1 s were selected to generate the test utterances by convolving the clean speech with different room impulse responses (RIRs). The simulated RIRs were constructed using the image method [46]. The detailed room size, speaker, and microphone positions are shown in Appendix B.

### 4.2. Results and Analysis

**Figure 8:**
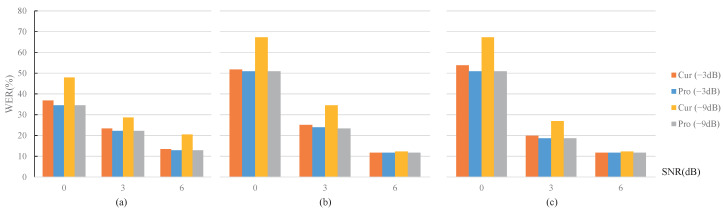
WER comparisons under different noise and SNR conditions. (**a**) White noise. (**b**) Babble noise. (**c**) Factory1 noise.

**Figure 9:**
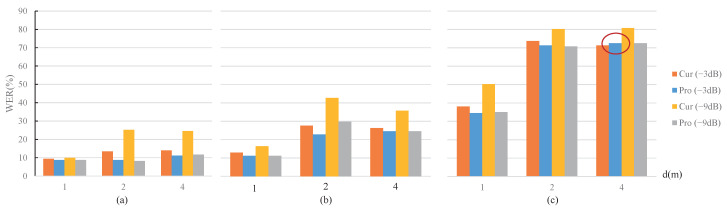
WER comparisons under different RT60 and speaker-microphone distance conditions. (**a**) RT60 = 0.5 s. (**b**) RT60 = 0.7 s. (**c**) RT60 = 1 s.

For the above-mentioned test utterances with noises or reverberations, the corresponding WER results of the ASR module are shown in Figure 8 and Figure 9, respectively. The legends “Cur” and “Pro” represent the current and the proposed strategies, respectively, and the legends “(−3 dB)” and “(−9 dB)” represent the gain. Since a smaller WER value corresponds to a higher recognition accuracy, a lower height of the histogram in Figure 8 and Figure 9 depicts a better performance.

#### 4.2.1. WER Analysis

The experimental ASR system’s word error rate (WER) corresponding to the clean test utterances without any attenuation and noise was 8.19%, and the lowest WER in the experiment was around 10%. From the overall trend of WER changes in Figure 8 and Figure 9, it can be seen that under two significant types of simulation conditions, WER performance variations basically covered the process from near the best to almost failure. Therefore, the experimental conclusions are comprehensive and representative.

In detail, the following conclusions can be obtained.

- The WER result increased with decreasing gains in all 18 sets of comparison test conditions in Figure 8 and Figure 9. This is because a lower gain control will result in a greater reduction in the quality of the test utterances;
- The proposed MOST gain control strategy showed advantages over the current strategies under the 18 sets of comparison test conditions in Figure 8 and Figure 9, except for the penultimate set indicated by the red circle in the right down position of Figure 9. The abnormal results corresponded to the conditions of RT60 = 1 s, d = 4 m, and the gain setting of −3 dB, within which the proposed MOST gain control strategy was slightly higher by 1%. The abnormal WER results exceeded 70%. The reason is likely that such a harsh reverberation condition is close to the working limitation of the experimental ASR system; thus, the WER performance of the ASR system is no longer positively related to distance;
- For a certain noise type shown in Figure 8, the power ratio of the clean speech to the selected noise is negatively correlated to the performance improvement of the proposed MOST gain control strategy.

#### 4.2.2. Absolute WER Reduction Analysis

**Figure 10:**
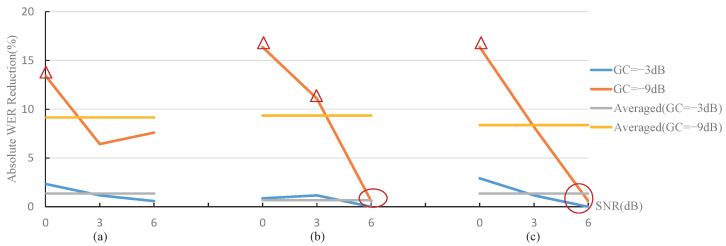
Absolute WER reduction under different noise and SNR conditions. (**a**) White noise. (**b**) Babble noise. (**c**) Factory1 noise.

**Figure 11:**
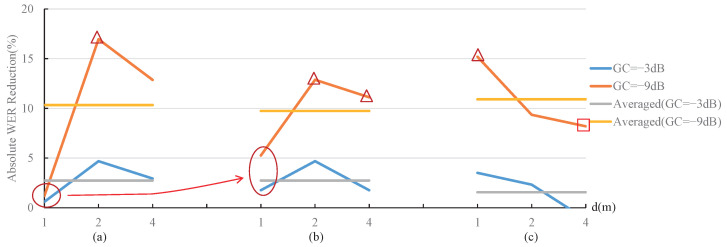
Absolute WER reduction under different RT60 and speaker-microphone distance conditions. (**a**) RT60 = 0.5 s. (**b**) RT60 = 0.7 s. (**c**) RT60 = 1 s.

In order to show the degree of improvement of the proposed MOST gain control strategy to the WER, the absolute WER reduction is calculated by

(7) $$W\;E\;R_{\mathrm{absolute}}\left(\%\right)={W\;E\;R}_{\mathrm{current}}-{W\;E\;R}_{\mathrm{MOST}}$$

where ${W\;E\;R}_{\mathrm{current}}$ and ${W\;E\;R}_{\mathrm{MOST}}$ represent the WER result corresponding to the current strategies and the proposed MOST gain control strategy, respectively. Obviously, a larger $W\;E\;R_{\mathrm{absolute}}$ represents a more remarkable WER performance improvement. The $W\;E\;R_{\mathrm{absolute}}$ results based on Figure 8 and Figure 9 are shown in Figure 10 and Figure 11. The legends “GC(−3 dB)” and “GC(−9 dB)” represent the $W\;E\;R_{\mathrm{absolute}}$ calculated under the gain of −3 dB and −9 dB, respectively. The legends “Averaged (GC = −3 dB)” and “Averaged (GC = −9 dB)” indicate the average value of three sets of $W\;E\;R_{\mathrm{absolute}}$ at −3 dB and −9 dB gain, respectively, and the three sets of $W\;E\;R_{\mathrm{absolute}}$ correspond to a specified noise type or RT60. The average values represented by the straight lines in Figure 10 and Figure 11 describe the average WER performance improvement under the specified conditions.

From Figure 10 and Figure 11, the following conclusions can be obtained.

- The gain control strategy dramatically influences the performance improvement degree of the proposed MOST gain control strategy, and the noise type, signal-to-noise ratio, RT60, and distance factors have a relatively small influence. The average performance improvement of the proposed MOST gain control strategy was around 10% under the −9 dB gain condition, while it was about 2% under −3 dB gain condition;
- The performance improvement degree of the proposed MOST gain control strategy is rather effective under a lower gain. In the case of −9 dB gain, 8 of the 18 sets of the proposed MOST gain control strategy offered a WER reduction of more than 10%, as shown with the △ symbols in Figure 10 and Figure 11;
- The proposed MOST gain control strategy has a smaller performance improvement if the utterances are less affected by the noises or reverberations. (1) The WER reduction is relatively small for the test utterances with high SNRs, corresponding to the two circle positions in Figure 10. The reason is that such a high SNR provides enough features for the test utterance signal to be recognized by ASR; thus, the current strategies and our MOST gain control strategy both obtained better WER results, as shown in the two right sets of results in Figure 8b,c. (2) The WER reduction is not obvious under weak reverberation conditions, as shown in Figure 11. When the distance is very close (1 m) and RT60 is small (0.5 s) (corresponding to the two circle positions in Figure 11), the improvement effect is not apparent; therefore, all strategies can obtain good WER results, as shown in the two left sets of results in Figure 9a,b;
- The performance improvement degree of the proposed MOST gain control strategy becomes evident as the reverberation condition becomes severe. The increase in the vertical distance of the circles in Figure 11 shows that, as increasing the adverse effects of the environment become more serious, the improvement effect of the proposed MOST gain control strategy gradually emerges;
- The performance improvement degree of the proposed MOST gain control strategy decreases under extremely severe reverberation conditions. When the distance is extremely long (4 m) and RT60 is large (1.0 s) (corresponding to the position of the box in Figure 11), the ASR system can hardly work normally, so the WER results of the current strategies and MOST gain control strategy are both very poor, as shown in the two right sets of the results in Figure 9c.

The overall averaged WER reductions of the proposed MOST gain control strategy under reverberation and noise conditions are shown in Table 1. The results in Table 1 represent the mean WER results corresponding to different reverberation and noise conditions.

Results in Table 1 are more comprehensive and representative. Whether reverberation or noise test utterances, at a relatively small gain attenuation amplitude (−3 dB), the performance improvement of the proposed MOST gain control strategy is small, about 2%. As the gain attenuation increases, such as −9 dB, the proposed MOST gain control strategy dramatically improves the ASR system performance.

In summary, the proposed MOST gain control strategy reduces the adverse effects of the gain control to the greatest extent and realizes a significant performance improvement of the ASR system, especially under harsh environments and with more significant gain attenuation. Although the proposed MOST gain control strategy improves the ASR performance in a different way compared with the existing methods based on algorithm or subsystem updating, the proposed MOST gain control strategy’s effect is rather considerable and efficient under a medium usage of gain control operation (such as gain ≤−9 dB).

## 5. Discussion

The experiments were conducted on the Meridian dataset; this is the limitation of this paper. However, the ’improvement’ of this paper involves the speech signal features. The proposed MOST gain control strategy ensures that more features are fed into the recognition algorithm. Thereby, higher recognition accuracy is achieved. Features are the common foundation for the speech recognition of Mandarin and other languages. Thus, the proposed MOST gain control strategy is probably applicable for the other languages. One future work is to prove the effectiveness of the proposed MOST gain control strategy with other languages.

Moreover, unlike directly improving the ASR of the algorithm layer, this paper indirectly improves ASR performance by optimizing the noise caused by the gain control in the signal layer. The signal layer is the basis of the algorithm layer, and the influence and processing of the signal layer on the original sound signal take precedence over the algorithm layer. Therefore, in the research of ASR, the importance of the signal layer cannot be ignored. In future work, other aspects of the signal layer that affects the performance of ASR systems should be investigated, such as dynamic range compression at the microphone, which is a variant of gain control.

## 6. Conclusions

Inappropriate gain control strategies cause an increase in the ASR WER, with respect to a performance degradation of the human–computer interaction system. In this paper, an optimal gain control strategy named MOST was proposed to minimize this adverse impact. Our primary contribution involved modeling the gain control strategy and theoretically prove that unconstrained gain control will cause the performance degradation of the ASR system using the noise figure theory. The second contribution theoretically demonstrates that the proposed MOST gain control strategy is the optimal gain control strategy for the ASR system. Finally, comprehensive comparison experiments under different conditions were conducted on the Mandarin dataset. For a −9 dB gain setting, the proposed MOST gain control strategy improved the WER performance of the experimental ASR system by an average of up to 10%. Such a considerable performance improvement shows that the proposed gain control strategy, as well as its corresponding modeling method, are effective in real ASR systems.

## Figures and Tables

**Table 1:** Averaged absolute WER reduction of the proposed MOST gain control strategy relative to the current strategies.

Category	Reverberation		Noise	
Gain (dB)	−3	−9	−3	−9
Averaged absolute WER reduction (%)	2.3	10.3	1.1	9.0

**Figure A1:**
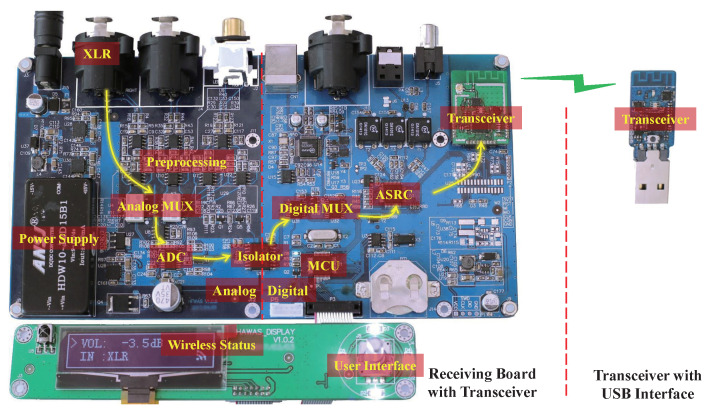
Wireless audio transmission system adopted in the ASR experiment.

**Figure A2:**
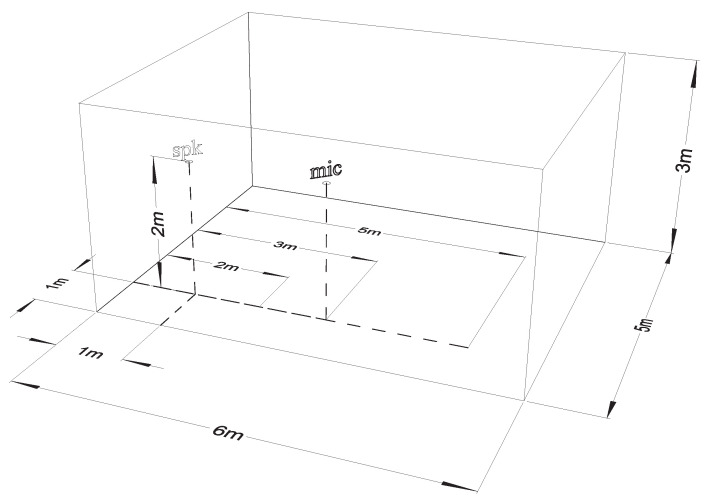
The room that is used to produce the experimental reverberations.

## Data Availability

Not applicable.

## Abbreviations

The following abbreviations are used in this manuscript:

ASR	automatic speech recognition
ACK	acknowledgement
ASRC	asynchronous sampling rate converter
AM	acoustic model
ADC	analog to digital converter
ANN	artificial neural network
aptX	audio delivers premium sound wirelessly via Bluetooth
aptX HD	aptX High Definition
aptX LL	aptX Low Latency
BT SBC	Bluetooth subband codec
DSP	digital signal processor/processing
DNN	deep neural network
FNN	fuzzy neural network
HMM	hidden Markov model
GUI	graphical user interface
GC	gain control
LM	language model
LNA	low noise amplifier
LDAC	a proprietary audio coding technology developed by Sony
MCU	micro control unit
MFCC	Mel-frequency cepstral coefficients
ML	machine learning
MOST	maximized original signal transmission
MUX	multiplexer
mic	microphone
NW	network
PP	preprocessing
PM	pronunciation model
RF	radio frequency
RNN	Recurrent neural network
RIR	room impulse responses
RT60	metric reverberation time 60
SVM	support vector machines
SOC	System on chip
spk	speaker
TDNN	time delay neural network
THCHS-30	an open Chinese speech database published at Tsinghua University
THD + N	total harmonic distortion and noise
VUI	voice user interface
WER	word error rate
*d*	speaker to microphone distance
*F*	noise figure
$F_{\mathrm{ideal}}$	noise figure of ideal gain control
$F_{\mathrm{actual}}$	noise figure of actual gain control
$F_{\mathrm{GC}}$	noise figure of gain control part of the basic unit model
$F_{\mathrm{ASR}}$	overall noise figure of ASR system
$F_{\mathrm{Pro}}$	noise figure of processing part of the basic unit model
$F_{1a},F_{2a},\ldots$	noise figure of left part of 1th, 2th, …basic unit model
$F_{1b},F_{2b},\ldots$	noise figure of right part of 1th, 2th, …basic unit model
$G_{\mathrm{req}}$	gain requirement in logarithmic form
GL	Linear gain
$GL_{\mathrm{GC}}$	linear gain of gain control part of basic unit model
$GL_{\mathrm{Pro}}$	linear gain of processing part of basic unit model
$GL_{1a},GL_{2a},\ldots$	linear gain of left part of 1th, 2th, …basic unit model
$GL_{1b},GL_{2b},\ldots$	linear gain of right part of 1th, 2th, …basic unit model
$P_{\mathrm{signal}}$	power of speech signal
$P_{\mathrm{noise}}$	power of noise
$P_{\mathrm{noisefloow}}$	power of the noise floor
SNR	Signal to noise ratio
$SNR_{\mathrm{input}}$	signal to noise ratio at the input port
$SNR_{\mathrm{output}}$	signal to noise ratio at the output port
$WER_{\mathrm{absolute}}$	absolute WER reduction
$WER_{\mathrm{current}}$	WER corresponds to current gain control strategy
$WER_{\mathrm{MOST}}$	WER corresponds to proposed MOST gain control strategy

## Appendix A. Experimental Hardware

The wireless audio transmission system used in the experiment is shown in Figure A1. The wireless audio transmission system we developed possesses the proposed MOST gain control strategy and can transmit audio signals with CD-quality. In detail, the wireless audio transmission system serves two purposes, as follows.

- The wireless audio transmission system demonstrates an application example of the proposed MOST gain control strategy;
- Parts of the wireless audio transmission system are adopted to simulate the user-end device in Figure 7 and transmission parts in the ASR experiment.

The transceiver in Figure A1 ensures the realization of the proposed MOST gain control strategy by using a data channel to transmit the gain control commands. It contains a CC8531 system-on-chip (SOC)-based 2.4 GHz transceiver and a 22 dBm RF power amplifier for extending the range. The gain control requirements are obtained through the remote control or the encoder of the receiving board with the transceiver. Then the gain control commands are forwarded by the MCU to the transceiver, and are sent to the transceiver with USB interface with audio signals simultaneously. Finally, the gain control operations are performed at the back-end.

The lines with the arrow in Figure A1 represent the direction of the audio signal flow used in the experiment. The audio interface box firstly received the speech signal from the computer through an XLR interface, buffered and filtered by low noise amplifiers OPA1632, and then output through a relay that switched balanced and unbalanced analog input signals. Secondly, the output speech signal was adjusted by the amplifier OPA1632 to match with the later unit dynamic range requirement, and quantized by the ADC chip CS5381. Finally, the speech signal was sent to the USB receiver equipped on computer 2 and handled by the ASR algorithm. The audio clock was resynchronized twice to decrease the jitter, and the digital and analog circuits of the audio interface box were designed to be isolated for a lower noise level. The basic parameters of the signal chain are as follows: (1) THD + N = −73.57 dB, SNR = 78.55 dB, (1 kHz, 0 dB input, band = 20 Hz–20 kHz, unweighted); (2) ADC (16-bit, 44.1 kHz). Moreover, considering that the over-the-air data rate and audio latency performance are essential indexes for ASR systems, comparisons of these indexes between our experimental platform and multiple mainstream Bluetooth-based wireless audio transmission scheme are shown in Table A1.

**Table A1:** Over-the-air data rate and audio latency performance comparison between our wireless audio transmission system and various Bluetooth-based technologies.

Index	BT SBC	aptX	aptX HD	aptX LL	LDAC	Ours’
Data rate/Mbps	0.328	0.384/0.325	0.576	0.352	0.99	5.0
Latency/ms	220	130	220	40	>80	10.7–40

## Appendix B. Experimental Reverberation Conditions

The room and position information of the speaker and microphone for the experimental reverberations are shown in Figure A2. It is assumed that the clean speech was received by an omnidirectional microphone placed in a rectangular room with dimensions [5 × 6 × 3] (m), and all six wall surfaces of the room have the same reflection coefficient. The speaker is at the position of [1, 1, 2] (m) represented by “spk”. Parameter “d” represents the speaker–microphone distance, and the microphone is placed at the position of [1, 2, 2] (m) for d = 1 m, [1, 3, 2] (m) for d = 2 m, and [1, 5, 2] (m) for d = 4 m, as shown by “mic”.
